# Relation of gait measures with mild unilateral knee pain during walking using machine learning

**DOI:** 10.1038/s41598-022-21142-2

**Published:** 2022-12-23

**Authors:** Kathryn L. Bacon, David T. Felson, S. Reza Jafarzadeh, Vijaya B. Kolachalama, Jeffrey M. Hausdorff, Eran Gazit, Neil A. Segal, Cora E. Lewis, Michael C. Nevitt, Deepak Kumar, Multicenter Osteoarthritis Study Investigators, David T. Felson, Neil A. Segal, Cora E. Lewis, Michael C. Nevitt

**Affiliations:** 1 Boston University Chobanian & Avedisian School of Medicine, 650 Albany Street, Suite X200, Boston, MA 02118 USA; 2 Tel Aviv University, Tel Aviv, Israel; 3 Tel Aviv Sourasky Medical Center, Tel Aviv, Israel; 4 University of Kansas Medical Center, Kansas City, USA; 5 University of Alabama at Birmingham, Birmingham, AL USA; 6 University of California, San Francisco, San Francisco, CA USA

**Keywords:** Osteoarthritis, Osteoarthritis, Pain, Rheumatology, Musculoskeletal system

## Abstract

Gait alterations in those with mild unilateral knee pain during walking may provide clues to modifiable alterations that affect progression of knee pain and osteoarthritis (OA). To examine this, we applied machine learning (ML) approaches to gait data from wearable sensors in a large observational knee OA cohort, the Multicenter Osteoarthritis (MOST) study. Participants completed a 20-m walk test wearing sensors on their trunk and ankles. Parameters describing spatiotemporal features of gait and symmetry, variability and complexity were extracted. We used an ensemble ML technique (“super learning”) to identify gait variables in our cross-sectional data associated with the presence/absence of unilateral knee pain. We then used logistic regression to determine the association of selected gait variables with odds of mild knee pain. Of 2066 participants (mean age 63.6 [SD: 10.4] years, 56% female), 21.3% had mild unilateral pain while walking. Gait parameters selected in the ML process as influential included step regularity, sample entropy, gait speed, and amplitude dominant frequency, among others. In adjusted cross-sectional analyses, lower levels of step regularity (i.e., greater gait variability) and lower sample entropy(i.e., lower gait complexity) were associated with increased likelihood of unilateral mild pain while walking [aOR 0.80 (0.64–1.00) and aOR 0.79 (0.66–0.95), respectively].

## Introduction

Individuals with knee osteoarthritis (OA) are known to exhibit multiple gait alterations. These may include alterations in spatio-temporal parameters (e.g., greater stride duration, lower cadence, lower gait speed, lower stride length) and in kinematics and kinetics (e.g., varus thrust, greater knee adduction moment)^1,2^. These alterations in gait, specifically greater knee adduction moment and varus thrust^3,4^, in people with knee OA can lead to accelerated disease progression ^5–9^, and hence, interventions to modify gait to slow progression of knee OA have been of significant clinical interest^10,11^.

While most prior studies have focused on gait alterations related to structural OA pathology^12,13^, less is known about their relation to knee pain^14^, particularly mild knee pain. It is known that cartilage loss in OA progression is not a direct source of pain in mild to moderate OA^15^. An indicator of early OA may be unilateral knee pain, hypothesized to alter loading patterns across both knees and contribute to the eventual development of OA in the contralateral knee. Most persons who start with unilateral knee pain from OA eventually develop bilateral OA. Thus understanding the associations between mild unilateral knee pain and gait may provide important mechanistic insights about gait in knee pain and OA, insights that could be used to identify interventions to modify gait to reduce pain or to prevent progression to more severe pain.

Prior gait studies in people with knee OA have additional limitations. Most have relied on small sample sizes, limiting their ability to comprehensively characterize gait and account for confounders. Also, prior studies selected a limited number of gait variables which could lead to important information being missed. Finally, prior studies used 3D optical motion capture to characterize gait. Although optical motion capture provides high accuracy, it is expensive and time consuming to collect and process these data, limiting its application to research laboratories^8^.

Advances in wearable movement sensors allows rapid assessment of gait in large cohorts. Few studies of OA or of knee pain have used wearable inertial sensors in “native” knees (i.e. no TKR)^16,17^. Furthermore, work in Parkinson’s disease^18–20^ has demonstrated the potential of state-of-the-art machine learning (ML) analytical techniques, particularly when combined with wearable inertial sensors for the collection of gait data, either in or outside gait labs. Machine learning techniques allow the use of the computer to “learn” connections within the data using few assumptions. These approaches, however, have not yet been systematically applied to gait in knee pain or OA^21,22^.

Our objective was to determine gait alterations associated with mild unilateral knee pain using gait data collected with inertial sensors in a large cohort of participants with or at risk of knee OA from, the Multicenter Osteoarthritis (MOST) study. We first used ML approaches to select gait parameters related to mild knee pain. We then determined the associations of these gait parameters with unilateral knee pain while adjusting for common covariates for pain and OA.

## Results

**Figure 1:**
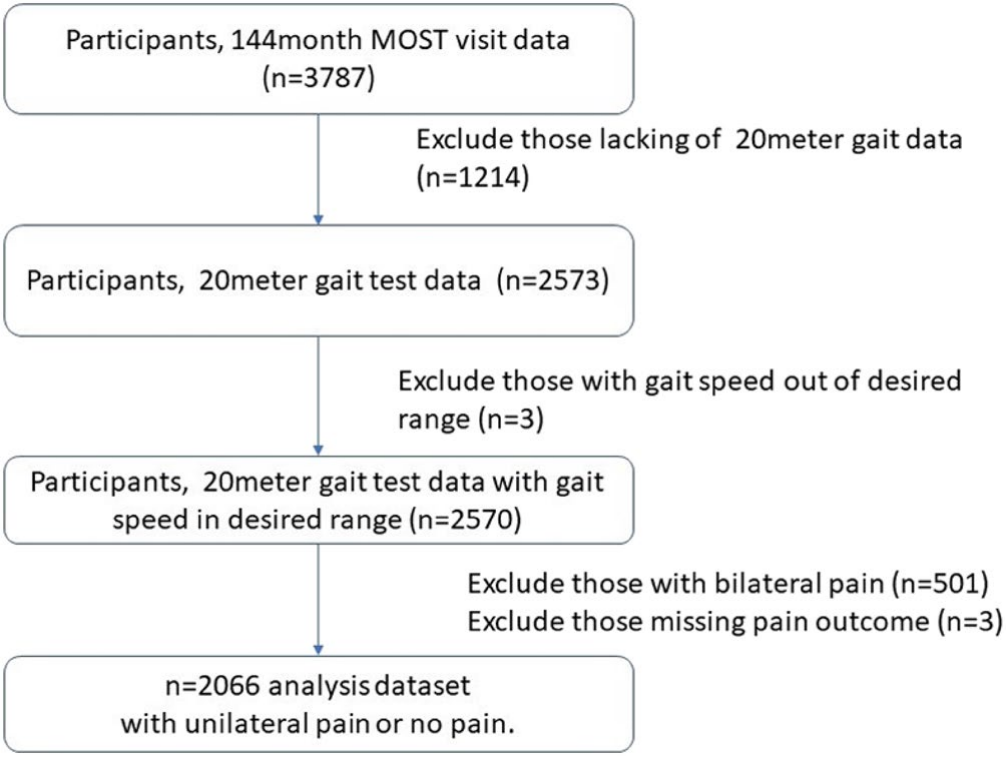
Selection for analysis dataset. Selection of participants for analysis dataset from MOST cohort.

Our study sample (Fig. 1, Table 1) included 2066 participants (mean age 63.3 [SD: 10.4] years, 56% female) from the 144-month visit of the MOST study. In this cohort, 21.3% (n = 440) of participants had unilateral mild knee pain while walking; 4.8% (n = 99) had moderate/severe unilateral knee pain while walking. Of those with unilateral walking pain, 15.0% (n = 81) had unilateral radiographic OA; 20.4% (n = 110) had bilateral radiographic OA. Thus although the focus of this analysis is on unilateral knee pain while walking, there are participants in this sample with bilateral radiographic OA.

**Table 1:** Participant Characteristics at 144-month MOST visit (baseline).

Characteristic	n = 2066
Age, years mean (sd)	63.3 (10.4)
Female, %	55.7
BMI, mean (sd)	29 (5.5)
Whites, %	84.5
CES-D Score (calculated from full twenty questions) mean (sd)	5.8 (6.5)
Radiographic knee OA, % categ: None/unilateral OA/bilateral OA	72.9/ 13.7/ 13.5 (n = 1446/ 271/ 268)
Unilateral knee pain walking, % categ: none/mild/moderate or severe	73.9/ 21.3/ 4.8 (n = 1527/ 440/ 99)
Gait speed, m/s mean (sd)	1.3 (0.2)

### Machine learning for variable selection

The median area under the receiver operating curve (AUC) across 100 runs was 0.75 (2.5–97.5th percentiles = 0.72–0.78). The top contributing gait parameters for unilateral knee pain based on the variables importance (VIM) statistic are shown in Table 2. Non-gait-related variables chosen by the ML process as “influential” include age, BMI, Center for Epidemiological Studies Depression (CES-D) Scale, and radiographic OA. These variables, plus sex and race/site, had already been chosen as confounders to be included in the adjusted models.

**Table 2:** Variables selected from machine learning.

Predictors selected from machine learning	Importance (% runs, variables found to be important)
BMI	100
CES-D	100
Radiographic OA	100
Gait Speed (m/s)	100
Age	99
Sample Entropy	99
Step Regularity	83
Amplitude Dominant Frequency (psd)	68
Step Symmetry	40
Left Swing Time (s)	35

In our testing for multicollinearity among model variables, there were no correlations greater than 0.80, no variables with tolerance values less than 0.10, and no variables with variable inflation factors greater than 10. Inspection of eigenvalues and condition values gave no indication of issues. Based on these collinearity diagnostics, we felt there was no evidence for collinearity sufficient to warrant dropping variables from the model.

### Gait alterations related to knee pain during walking

Among those with mild unilateral knee pain during walking (n = 440), lower step regularity and lower sample entropy, but not gait speed, were related to greater odds of pain (Table 3) in both unadjusted and adjusted models. The unadjusted model included only gait-related variables as we consider these collectively to be the “exposure”.

**Table 3:** Associations with unilateral mild pain while walking.

Predictors selected from machine learning	Unadjusted Mild pain versus no pain OR (95% CI)^ad^	Adjusted Mild pain versus no pain aOR^d^ (95% CI)^bd^
Gait Speed (m/s)^c^	1.03 (0.84–1.27)	1.16 (0.92–1.46)
Sample Entropy^c^	0.83 (0.70–0.99)	0.79 (0.66–0.95)
Step Regularity^c^	0.78 (0.64–0.95)	0.80 (0.64–1.00)
Amplitude Dominant Frequency (psd)^cd^	0.94 (0.79–1.11)	0.95 (0.79–1.14)
Step Symmetry^c^	1.02 (0.89–1.17)	0.98 (0.85–1.14)
Left Swing Time^c^ (s)	0.90 (0.80–1.02)	0.98 (0.85–1.12)

^a^Model 1: Unadjusted model including only gait variables, listed above.

^b^Model 2: Adjusted for age, race, site, sex, BMI, CES-D, and radiographic OA status of ipsilateral and contralateral knees.

^c^All continuous variables are standardized.

^d^*aOR *adjusted odds ratio,* CI* confidence interval*, psd* power spectral density.

The sensitivity analysis with the 3-category pain outcome including those with moderate or severe unilateral pain during walking (Table 4) largely confirmed the findings of our primary analysis. However, among those with moderate or severe unilateral knee pain during walking (n = 99), lower gait speed, but not sample entropy or gait regularity, was associated with greater odds of pain. An additional sensitivity analysis excluding those with gait speed greater than 1.8 m/s gave essentially the same results as our primary analyses.

**Table 4:** Associations with unilateral pain while walking—sensitivity analysis with 3-category pain outcome.

Predictors selected from machine learning	Mild pain versus no pain aOR^c^ (95% CI)^a c^	Moderate/severe pain versus no pain aOR^c^ (95% CI)^a c^
Gait Speed (m/s)^b^	1.16 (0.92–1.46)	0.63 (0.39–1.01)
Sample Entropy^b^	0.79 (0.66–0.95)	0.92 (0.65–1.30)
Step Regularity^b^	0.79 (0.64–0.98)	0.91 (0.60–1.39)
Amplitude Dominant Frequency (psd)^b c^	0.95 (0.79–1.14)	1.31 (0.91–1.87)
Step Symmetry^b^	0.99 (0.85–1.15)	0.84 (0.62–1.14)
Left Swing Time^b^ (s)	0.98 (0.85–1.13)	0.86 (0.66–1.11)

^a^Adjusted for age, race, site, sex, BMI, CES-D, and radiographic OA status of ipsilateral and contralateral knees.

^b^All continuous variables are standardized.

^c^*aOR* adjusted odds ratio, *CI* confidence interval, *psd* power spectral density.

## Discussion

In this cross-sectional analysis of a community-dwelling population with or at risk of knee OA, we observed that lower step regularity (i.e., greater gait variability) and lower sample entropy (i.e., lower gait complexity) were related to greater odds of mild unilateral knee pain. Using a large cohort and robust machine learning approaches, our results provide information on gait alterations that may be specifically related to mild knee pain during walking. It is of note that the majority of this sample did not have severe radiographic OA; they had walking pain in what is likely early OA. Given the age of this sample, work by Cibere et al.^23^ using MRIs has shown many persons in this age range with chronic knee pain have early OA even if it is not seen in x-rays.

It is challenging to compare the results from our work with prior studies given the paucity of research on gait alterations in those with mild knee pain. The studies available focused on kinematic and kinetic gait parameters^14,24–27^ and report conflicting findings on the relation of measures of knee joint loading (e.g., knee adduction moment) and severity of knee pain.

Lower step regularity reflects greater variability of the center of mass motion. In the absence of a significant difference in stride regularity, lower step regularity also reflects asymmetry of center of mass motion during gait^28^. While measures of gait variability have been reported to be sensitive to mild to moderate gait impairments in people with neurological impairments (for example, Parkinson’s disease and multiple sclerosis)^29,30^, evidence exists for lower step regularity in people with knee OA^31,32^. Our findings suggest altered neuromotor control of center of mass motion in the presence of mild unilateral knee pain during walking and may reflect an early adaptation of the nervous system to knee pain. This hypothesis aligns with theories^33–35^ suggesting a link between neurobiological mechanisms underlying chronic pain and control of movement. Interventions to improve gait symmetry in individuals with mild pain may improve step regularity and could be investigated in future studies^36^.

The idea of altered neuromotor control in the presence of pain is further supported by our finding of lower sample entropy (i.e., lower gait complexity) being related to mild pain.. Importantly, the association of sample entropy with knee pain was present after adjusting for age given that with aging, gait becomes less complex^37^. Hence, the association of knee pain with sample entropy may reflect changes in neuromotor control beyond those due to aging. Both greater variability and lower complexity are considered detrimental gait adaptations and are associated with worse mobility outcomes including greater fall risk^29,30,38^. Lower gait complexity indicates reduced adaptability of neuromotor control to external perturbations and may reflect increased attention to pain and has been found in persons with OA who have knee buckling episodes^39,40^. Interventions to increase gait adaptability or to reduce attention to pain (e.g., mindfulness meditation) could increase gait complexity in people with knee OA^41–43^.

While a few spatio-temporal measures of gait (e.g., swing time) were highlighted in the machine learning model, they were not found to be significant in the logistic regression model. This may be because they were related to covariates added later such as age and BMI or because their relation to mild knee pain was mathematically complex and not captured well by our logistic regression approach.

Given the cross-sectional nature of our study, it is not possible to determine the direction of causality. For example, individuals with knee pain may adapt their gait patterns in response to pain and thereby reduce their pain during walking^25,44^. Our findings of lower step regularity and complexity may reflect these adaptations. This is supported by findings of reduction in gait variability after administration of an opioid analgesic in people with knee OA^45^. Longitudinal studies are needed to further understand the associations between mild pain and neuromotor control of gait. If these studies show that step regularity and complexity contribute to pain and mobility limitations, they could be targets of therapeutic interventions.

In sensitivity analyses, we confirmed the primary findings of lower step regularity and lower sample entropy being related to greater odds of unilateral mild knee pain during walking. In our analyses of moderate-severe knee pain versus no pain during walking, slower gait speed emerged as important factor. This latter finding aligns with prior studies reporting reduced walking speed in people with advanced structural knee OA^1^. Although our results are cross-sectional, slow gait speed in older adults may have long-term consequences such as increased disability, morbidity, and mortality^46–48^. With greater pain severity, individuals may adopt the simplest strategy to reduce joint loading i.e., reducing gait speed. Another possibility is that brain function of different regions may be altered in those with moderate-severe pain. In older adults, the ability to sustain attention has been reported to be related to gait variability, whereas executive functioning is related to gait speed^49^. Hence, in people with moderate-severe pain, brain regions related to executive functioning may exhibit altered function, whereas in people with mild pain, brain regions related to attention are altered. Longitudinal studies are needed to confirm these cross-sectional observations. An additional sensitivity analysis excluding those with gait speed greater than 1.8 m/s gave essentially the same results as our primary analyses.

The MOST study has several key strengths for this type of analysis: it is a large community-dwelling cohort of men and women with data on risk factors and characteristics of OA, and gait data from inertial sensors. We were able to evaluate people with mild pain. There were also limitations to our study. This is a cross-sectional analysis, so we cannot rule out reverse-causation. Also as in any observational study, residual confounding may exist.

One important strength of our study was the use of wearable sensors which allowed efficient collection of complex data from a large number of persons. Advances in wearable sensors and computing could allow for rapid and easy assessment of these gait outcomes in clinical and real-world settings^50^. These gait alterations, if shown to be important in longitudinal studies of knee pain, could provide clues about interventions to reduce pain in people with knee OA.

In summary, in this cross-sectional study of persons with or at risk of knee OA, measures of step regularity and complexity, derived using wearable inertial sensors, are altered in those with mild unilateral knee pain and may provide new insights into gait abnormalities that occur even with mild pain and that may have implications for strategies to use to prevent mild pain from progressing.

## Methods

### Study population

The Multicenter Osteoarthritis (MOST) study is an NIH-funded cohort study of men and women between 45 to 90 years of age at risk of knee osteoarthritis. Study participants were recruited in Birmingham, Alabama and Iowa City, Iowa^51^. The study started in 2003 when study participants were interviewed by telephone and attended clinic visits and participants with or at risk of knee OA were recruited. Further details of inclusion and exclusion criteria have been published^51,52^. In addition to the original cohort, a new cohort of 1500 persons with at most mild radiographic OA and moderate knee pain was recruited at the 144-month clinic visit (2016–2018) of the MOST study. All participants in the 144-month clinic visit (original and new cohort) underwent weight bearing posteroanterior (PA) and lateral fixed flexion radiographs, filled out the Western Ontario and McMaster Universities Osteoarthritis Index (WOMAC)^53^ and Center for Epidemiological Studies Depression (CES-D) Scale surveys and had weight and height measured according to the MOST protocol.

Combining the original cohort with the newly recruited one at the 144 month clinic visit, we carried out a cross-sectional analysis comparing individuals with unilateral knee pain while walking to those without knee pain while walking. We included participants who had inertial sensor gait data from a 20-m walk test at the 144-month clinic visit, self-report data on the WOMAC questions on pain while walking, and a score for OA severity based on the Kellgren and Lawrence (KL)^54^ grade.

#### Gait parameters

An inertial sensor system (OPAL, APDM Inc) was used to collect spatial and temporal measures during over-ground walking. During their 144-month clinic visit, participants completed two trials of a 20-m walk test in an obstacle-free laboratory setting, during which they wore inertial sensors on their trunk and bilateral ankles. Gait parameters available from the MOST database included spatiotemporal features and measures of gait symmetry, variability, and complexity (Table 5). These variables were extracted from the raw vertical acceleration signal from the trunk sensor using published algorithms. For each gait parameter for a participant, the mean of the two trials was used in the analyses^55^. The analysis sample was restricted to those participants with baseline gait speed in the range 0.3 to 2.3 m/second to exclude potentially invalid gait speed measurements (Fig. 1).

#### Pain while walking

We used the item asking about pain during level walking from the Western Ontario and McMaster Universities Osteoarthritis Index (WOMAC)^53^ Pain survey. Participants rated the pain during walking over the past 30 days in their right and left knees (separately) on a Likert scale (none, mild, moderate, severe, extreme). Because we were interested in gait changes that were likely to be asymmetrical, participants who reported bilateral pain while walking were excluded (Fig. 1). We then created a per-person unilateral pain outcome from the left and right knee values for each person (i.e. if an individual has pain, it will be in only one of the legs and these values can map directly into the per-person variable). For the primary per-person dichotomous pain outcome, participants were categorized into those with no pain during walking, versus those with mild unilateral pain during walking (n = 1967); participants with greater than mild pain are marked missing for this outcome. A 3-category pain outcome was then created for a sensitivity analysis including those with more pain, which grouped participants into (1) those with no pain while walking, (2) mild unilateral pain while walking, or (3) moderate or severe unilateral pain while walking (total n = 2066).

### Covariates

Age at baseline in years, and BMI (weight kg/ height m^2^) were used as continuous variables. Depressive symptoms were measured by the CES-D^56^ (range 0–60). A categorical covariate was created to combine study site (Iowa or Alabama sites) and race.

#### Radiographic OA status

We created a per-person radiographic OA status indicating the number of knees with a Kellgren-Lawrence (KL)^54^ score of 2 or greater: 0, 1 or 2 knees. According to the MOST protocol, once participants developed KL scores of 3 or greater or had a TKR, they no longer were eligible to obtain x-rays on that knee. KL scores from the 144-month MOST visit, if missing, were back-filled with a non-missing value from the most recent prior exam with a non-missing value, if available.

### Machine learning process for variable selection

**Figure 2:**
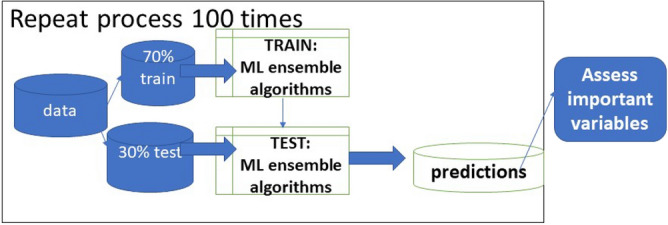
Machine learning process. Representation of the machine learning process repeated 100 times: data is split into 70% training set, 30% test set. Ensemble of ML algorithms is trained on the training set, then testing is done on the testing set and predictions are evaluated. Influential variables are assessed and saved for that run. At the end of all runs influential variables across all runs are assessed.

Our goal was to identify gait characteristics which may be associated with mild unilateral knee pain. As a first step towards this goal, we used an ensemble ML technique (“super learning”)^57,58^ as a feature reduction approach to identify important variables associated with the presence/absence of unilateral knee pain during walking. Super learner uses a multi-fold cross-validation to select the optimal combination of algorithms^58,59^ that theoretically achieves an accuracy superior to any single ML method. Our super learner configuration with fivefold cross-validation included a stacked ensemble^58,60^ of the following individual algorithms^58^ appropriate for binomial outcomes: discrete Bayesian additive regression trees, xgboost: extreme gradient boosting, generalized linear models (GLM) with convex penalties (that consisted of least absolute shrinkage and selection operator [LASSO], GLM ridge regression, and GLM elastic net, logistic regression, random forest(ranger: Fast(er) Random Forests)^61^, and support vector machine^62,63^. Variables included for possible selection by the super learning process included all inertial sensor gait variables, age, BMI, CES-D, sex, and radiographic OA status. Missing values were first imputed in the data used for the ML process, using multivariate imputation by changed equations (MICE)^64^ and data were then randomly split into 70% training (development) and 30% test (evaluation) sets. To increase robustness, the random data split and model training and testing were repeated 100 times as part of the ML process (see Fig. 2). A variable importance measure (VIM) statistic based on loss squared error^58^ identified variables which contributed to the prediction of unilateral knee pain in each run; we then took the 10 variables most frequently identified across 100 runs to use in logistic regression models. The area under the curve (AUC) was calculated for each run, and the median AUC across all runs was calculated.

### Logistic regression for direction and magnitude of association between gait variables and knee pain

The second and final step in our process to determining the direction and magnitude of association between gait variables and mild unilateral knee pain during walking was a logistic regression model built using the variables chosen by the ML process. Continuous variables were standardized. As our “exposure” is the set of gait variables, the unadjusted model shown (Table 3) includes the gait variables chosen with the ML process. Confounders found to be associated with knee pain in other studies, including age, sex, race, BMI, depressive symptoms, and person-level radiographic OA status were included in adjusted models, even if they were not selected by the ML process.

We evaluated the model variables for collinearity by reviewing correlations, tolerance values, and variable inflation factors as follows^65^. We first checked correlations on all variables, looking for any which might be greater than 0.8. In the case of a high correlation between model variables we would choose to drop (one or more) variables. We then examined tolerance values, where a value over 0.10 could indicate multicollinearity issues, and evaluated variable inflation factors to ensure that all were less than 10 for our model variables. Last we examined eigenvalues and condition values in the collinearity diagnostics from SAS. For any indication of multicollinearity we would choose to drop one or more variables and test the remaining variables again (Table 2).

**Table 5:** Gait variables available in the MOST database.

Variable	Explanation
Gait speed (m/s)	Total distance walked/total walking time
Cadence (steps/minute)	Number of steps taken per minute^66^
Step Length (m)	Total distance walked/number of steps taken
Stride Time (s)	Mean duration of gait cycle (i.e., stride)
Left Stance Time (s)	Mean duration of stance phase for left and right legs
Right Stance Time (s)	
Left Swing Time (s)	Mean duration of swing phase for left and right legs
Right Swing Time (s)	
Left stance percent (%)	Mean duration of stance phase for left and right legs expressed in percentage of the total gait cycle
Right stance percent (%)	
Left swing percent (%)	Mean duration of swing phase for left and right legs expressed in percentage of the total gait cycle
Right swing percent (%)	
Step symmetry	Expresses the symmetry of the acceleration between left and right limbs^28,67^
Gait asymmetry	Calculated as—100*|ln(Right Swing Time/Left Swing Time)|. Values of 0.0 reflect perfect symmetry and higher values reflect greater degrees of asymmetry^68^
Stride time coefficient of variation (CV)	Reflects the magnitude of the stride-to-stride variability of stride time^29^
Left stance time CV	Reflects the magnitude of the stride-to-stride variability of the left or right stance time
Right stance time CV	
Step regularity	Measure of regularity of the vertical acceleration measured from the trunk sensor between consecutive steps using autocorrelation. Low step regularity indicates that there is a low regularity between steps or a systematic asymmetry between left and right leg^28,67^
Stride regularity	Expresses the regularity of the vertical acceleration measured from the trunk sensor between consecutive strides using autocorrelation^28,67,69^
Sample entropy	Entropy is a measure that quantifies regularity in time series: the more predictable and less complex a series is, the lower the entropy value^70,71^
Phase coordination index (PCI)	A measure of bilateral coordination of gait assessed by quantifying the phase relationship between the step timing of the left and right legs^72^
Phase CV	Coefficient of variation of the phase^72^
Phase absolute difference	Measure of how close the phases are to 180°
Dominant frequency	Frequency with the largest amplitude in the power spectral density in the 0.5–3 Hz band of the vertical acceleration signal from the trunk sensor^73^
Amplitude of dominant frequency	The peak of the power spectral density of the vertical acceleration signal from the trunks sensor^73^
Width of dominant frequency	Width of the peak at half of its peak amplitude^73^
Lyapunov exponent (short divergent)	Estimate of local stability during gait^74^

For sensitivity analyses using the 3-category walking pain outcome, we first tested for proportional odds. As this assumption did not hold, we used multinomial logistic regression with a generalized logit model to evaluate the 3-category walking pain outcome with the same variables used in the dichotomous model. We also tested our models in an analysis sample further restricted on gait speed (gait speed < 1.8 m/s).

Analyses were performed using SAS software version 9.4 and R version 4.0.2.

### Ethics approval

All participants underwent an informed consent process approved by the Institutional Review Board Committee on Human Research at the participating institutions as listed in the Acknowledgements statement. Written consent is obtained from all participants at the beginning of a clinic visit at the clinical center. The consent covers all data collection scheduled for each grant cycle. Verbal consent is obtained for telephone interviews. Participants give written permission for clinical centers to obtain medical records needed for documentation of joint replacement surgery. There were no participants under age 16.

## Data Availability

All data used in this project is publicly available through the Multicenter Osteoarthritis Study (MOST), now at the NIA AgingResearchBiobank. Multicenter Osteoarthritis Study (nih.gov) is at: https://agingresearchbiobank.nia.nih.gov/studies/most/.
